# 7,8-Dihydroxycoumarin inhibits A549 human lung adenocarcinoma cell proliferation by inducing apoptosis via suppression of Akt/NF-κB signaling

**DOI:** 10.3892/etm.2013.1054

**Published:** 2013-04-09

**Authors:** YUE WANG, CHANG-FENG LI, LI-MING PAN, ZHONG-LI GAO

**Affiliations:** 1 Departments of Thoracic Surgery, China-Japan Union Hospital of Jilin University, Changchun, Jilin 133033;; 2 Endoscopy, China-Japan Union Hospital of Jilin University, Changchun, Jilin 133033;; 3 Department of Pediatrics, The First Bethune Hospital of Jilin University, Changchun, Jilin 130021, P.R. China

**Keywords:** 7,8-dihydroxycoumarin, lung adenocarcinoma, apoptosis, signaling, Akt, NF-κB

## Abstract

The Akt/NF-κB pathways are involved in numerous anti-apoptotic and drug-resistance events that occur in non-small cell lung cancer (NSCLC). In the present study, the role of 7,8-dihydroxycoumarin in the regulation of the anti-apoptotic Akt and NF-κBp65 signaling pathways was explored. A549 human lung adenocarcinoma cells were exposed to 7,8-dihydroxycoumarin with a final concentration of 25, 50 and 100 *μ*mol/l for 48 h. Quantitative polymerase chain reaction (PCR) and western blotting were performed to detect mRNA and protein expression, respectively. The MTT assay was performed to detect cell proliferation. The results demonstrated that anti-apoptotic phospho-Akt1 (pAkt1), phospho-IκBα (pIκBα), NF-κBp65 and Bcl-2 were inhibited and pro-apoptotic caspase-3 was upregulated in a concentration-dependent manner. At a concentration of 100 *μ*mol/l, the anti-apoptotic NF-κBp65 and Bcl-2 mRNA expression levels decreased 0.12 (5.82/48.5, treated/control)-fold and 0.17 (6.7/39.4, treated/control)-fold, respectively. The pro-apoptotic caspase-3 mRNA was upregulated 4.43 (39.4/8.9, treated/control)-fold. The anti-apoptotic pAkt1, pIκBα, NF-κBp65 and Bcl-2 proteins were downregulated, with blot grayscale values of 7.3 (vs. 52.4 control), 4.3 (vs. 42.2 control), 5.08 (vs. 44.5 control) and 5.92 (vs. 38.5 control), respectively. The proapoptotic caspase-3 was upregulated to a blot grayscale value of 27.8 (vs. 5.8 control). The proliferative activity of A549 cells was reduced significantly compared with that of the control cells (83.7, 27.2 and 9.5 vs. 100%, respectively; P<0.05 for each). 7,8-Dihydroxycoumarin plays an important role in the induction of apoptosis via suppression of Akt/NF-κB signaling in A549 human lung adenocarcinoma cells in a concentration-dependent manner. 7,8-Dihydroxycoumarin may be a candidate naturally-occurring drug for the treatment and prevention of lung adenocarcinoma.

## Introduction

Naturally occurring coumarins, a group of plant-derived polyphenolic compounds, serve as antimitotic, immunomodulating, antiviral, anticancer and cytotoxic agents in humans (1,2). A coumarin derivative, 7,8-dihydroxycoumarin, an active plant lactone extracted from Daphne Korean Nakai (3), is mainly used as an analgesic, antibacterial and antiviral agent, as well as to prevent and treat liver fibrosis in the clinic (1).

7,8-Dihydroxycoumarin and analogs have demonstrated significant antitumor effects and promote tumor apoptosis (4–7) via multiple signaling pathways. Elinos-Báez *et al* reported that 7-hydroxycoumarin inhibits anti-apoptotic Bcl-2 expression in lung cancer cells and promotes the expression of pro-apoptotic Bcl-2-associated X protein (Bax) (8). Other studies identified that coumarin is able to induce cervical and colon cancer cell apoptosis by activating the mitochondrial pathway and the caspase-3-dependent apoptotic pathway, to downregulate the anti-apoptotic NF-κB, Bcl-2 and Bcl-xL, and upregulate caspase-3 to promote the release of cytochrome (cyt) *c*(9,10). The coumarin derivative psoralidin is also able to enhance the role of tumor necrosis factor-related apoptosis-inducing ligand (TRAIL) in promoting the apoptosis and necrosis of HeLa cervical cancer cells (11). Rasul *et al* reported that the coumarin derivative xanthoxyletin induces S phase arrest and apoptosis in SGC-7901 gastric cancer cells (12). Bhattacharyya *et al* demonstrated that 7-hydroxy-6-methoxycoumarin induces the downregulation of aryl hydrocarbon receptor (AhR), CYP1A1, proliferating cell nuclear antigen (PCNA), Stat-3, survivin, matrix metalloproteinase (MMP)-2, cyclin D1 and c-myc, and upregulation of p53, caspase-3 and tissue inhibitor of metalloproteinases (TIMP)-2 (13). Singh *et al* reported a coumarin derivative (RKS262) that inhibits the ovarian cancer cell cycle and promotes apoptosis in cancer cells (14). Additionally, the authors identified that the coumarin derivative upregulates pro-apoptotic proteins Bid and Bok and inhibits anti-apoptotic Bcl-xL and Mcl-1, independently of pro-apoptotic mitogen-activated protein kinase (MAPK) p38 and stress-activated protein (SAP)/c-Jun N-terminal kinase (JNK) activation. Bhattacharyya *et al* reported that coumarin enhances pro-apoptotic p53, PCNA, Bad, Bax, apoptotic protease activating factor (Apaf), cyt *c,* caspase-3 and caspase-9 expression in melanoma (skin cancer) cells, and inhibits the anti-apoptotic factors Akt, Bcl-2, Bcl-xL and NF-κB (15). Thati *et al* also identified that coumarin derivatives enhance the malignancy of pro-apoptotic factors caspase-3 and -9 (16).

The main types of lung carcinoma include small-cell lung cancer (SCLC) and non-small-cell lung cancer (NSCLC); lung adenocarcinoma accounts for 40% of NSCLCs (17,18). Lung adenocarcinoma cells overexpress multiple anti-apoptotic signals. The Akt/NF-κB pathways are involved in a number of anti-apoptotic and drug-resistant events that occur in lung adenocarcinoma (17,18). Therefore, we hypothesize that 7,8-dihydroxycoumarin may also play an important role in promoting the apoptosis of lung adenocarcinoma cells by suppressing the Akt and NF-κB signaling pathways.

In the present study, 7,8-dihydroxycoumarin was administered to lung adenocarcinoma cells to investigate its effect on the apoptotic signaling pathways.

## Materials and methods

### Materials

**Figure 1:**
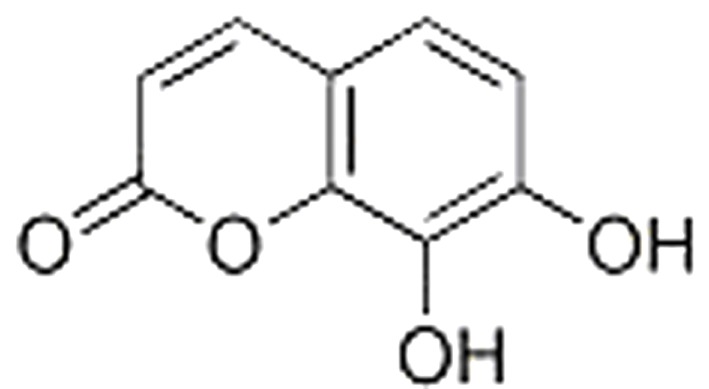
Structure of 7,8-dihydroxycoumarin.

7,8-Dihydroxycoumarin (purity, 99.6%; Tauto Biotech Ltd. Co., Shanghai, China) was dissolved in 0.9% NaCl solution, followed by filtration with a 0.02-mm filter (Millipore, Billerica, MA, USA). The structure of 7,8-dihydroxycoumarin is shown in Fig. 1. A total protein extraction kit and a TRIzol total RNA extraction kit were purchased from Invitrogen Life Technologies (Carlsbad, CA, USA). The anti-phospho-IκBα (phospho S32/S36; sc-8404), anti-NF-κBp65 (sc-8008), anti-Bcl-2 (sc-509) and anti-caspase-3 (sc-7272) antibodies were purchased from Santa Cruz Biotechnology, Inc. (Santa Cruz, CA, USA). The anti-phospho-Akt1 (phospho T308; ab105731) and anti-glyceraldehyde 3-phosphate dehydrogenase (GAPDH; ab8245) antibodies were purchased from Abcam (Beijing, China); these antibodies were mouse monoclonal. The horse-radish peroxidase (HRP)-labeled goat anti-mouse secondary antibody was purchased from Abcam. 3-(4,5-Dimethylthiazol-2-yl) 2,5-diphenyltetrazolium bromide (MTT) was purchased from Sigma (St. Louis, MO, USA). The Moloney murine leukemia virus reverse transcriptase (M-MLV RTase) kit was purchased from Promega Corporation (Shanghai, China). The 2X SYBR real-time polymerase chain reaction (PCR) kit was purchased from Roche Diagnostics (Shanghai, China). The bicinchoninic acid (BCA) protein detection kit and enhanced chemiluminescence (ECL) detection kit were purchased from Pierce Chemicals, Thermo Fisher Scientific Inc. (Rockford, IL, USA).

### Cell line

The A549 human lung adenocarcinoma cell line was purchased from American Type Culture Collection (ATCC no. CCL-185; Manassas, VA, USA). The cells were cultured in Dulbecco’s modified Eagle’s medium (DMEM) containing 10% fetal bovine serum (Gibco, Invitrogen Life Technologies) in a 5% CO_2_ incubator and passaged with 0.25% trypsin (Sigma, Ronkonoma, NY, USA) and 0.03% ethylenediamine tetraacetic acid (EDTA) solution.

### Treatment

The A549 cells were digested, suspended and seeded into each well of six-well plates with a density of 1.0×10^6^ cells/ml in 2 ml complete culture medium. The cells were cultured for 24 h and then exposed to 7,8-dihydroxycou-marin for 48 h. 7,8-Dihydroxycoumarin was dissolved in 0.9% NaCl solution and added to cells, forming a final concentration of 25, 50 and 100 *μ*mol/l. Equivalent 0.9% NaCl solution was added to cells as the control.

### Quantitative PCR (qPCR)

The A549 cells were harvested and total RNA was extracted with the total RNA extraction kit using the TRIzol method. The first strand cDNA was synthesized using M-MLV RTase according to the manufacturer’s instructions. Real-time PCR was performed using the cDNA template according to the manufacturer’s instructions. Amplification of GAPDH was used as an inner control in each reaction system. The reaction conditions were as follows: 40 cycles of 95°C for 30 sec, 58°C for 60 sec and 72°C for 60 sec. The primers were designed based on the Genbank sequence using Beacon Designer 7 (PREMIER Biosoft, Palo Alto, CA, USA). Primer synthesis and DNA sequencing were performed by Shanghai Sangon (China). The primer sequences were as follows: NF-κBp65, sense: 5′-GCAAAGGAAACGCCAGAAGC-3′ and antisense: 5′- CACTACCGAACATGCCTCCAC-3′; Bcl-2, sense: 5′-ATGACTTCTCTCGTCGCTACT-3′ and antisense: 5′-CCCATCCCTGAAGAGTTCCGA-3′; caspase-3, sense: 5′-CATGGCCTGTCAGAAAATAC-3′ and antisense: 5′-TAACCCGAGTAAGAATGTGC-3′; GAPDH (housekeeper gene), sense: 5′-AATGTGTCCGTCGTGGATCTG-3′ and antisense 5′-CAACCTGGTCCTCAGTGTAGC-3′.

### Western blotting

Western blotting was used to detect the protein expression of phospho-Akt1 (pAkt1), phospho-IκBα (pIκBα), NF-κBp65, Bcl-2 and caspase-3. The A549 cells were harvested and cell lysis was performed using the eukaryotic cell lysis buffer followed by extraction of total protein, according to the manufacturer’s instructions (Beyotime Inst. Biotech., Nanjing, Jiangsu, China). Protein quantity was determined by a BCA method. Using 30 *μ*g for each sample, proteins were separated by 12% sodium dodecyl sulfate polyacrylamide gel electrophoresis (SDS-PAGE) and were blotted with a wet transfer device (Bio-Rad, Shanghai, China) to a nitrocellulose membrane. The membrane was then immersed in blocking solution containing 10% skimmed milk in phosphate-buffered saline (PBS) Tween-20 (PBST), followed by agitation for 1 h. After washing three times with Tris-buffered saline Tween-20 (TBST) for 5 min each, the membrane was immersed in primary antibody diluted to 1:1,000 in the blocking solution at room temperature and then agitated for 1 h. After washing again, the membrane was incubated in HRP-labeled secondary antibody diluted to 1:10,000 in blocking solution at room temperature and then agitated for 1 h. After another rinse, the membrane underwent color development by an ECL method, followed by X-film photography. GAPDH protein was used as an inner control. The gray scale values (total raw density) of blots were measured with the VisionWorksLS analysis software available in the UVP EC3 (600) Imaging System (UVP, LLC, Upland, CA, USA).

### MTT assay

After 48 h, the medium was refreshed to discard the 7,8-dihydroxycoumarin. Cells were supplemented with 200 *μ*l MTT solution (5 mg/ml), followed by incubation in a CO_2_ incubator for another 4 h. The supernatant was discarded and each well was supplemented with 500 *μ*l dimethylsulfoxide (DMSO; Sigma). When the purple crystals at the bottom of the well were completely dissolved, the absorbance value was measured with a Thermo Multiskan MK3 microplate reader (Thermo Fisher Scientific Inc., Waltham, MA, USA) at a wavelength of 490 nm. Cell viability (%) = experimental absorbance/normal absorbance ×100.

### Statistical analysis

Data are presented as means ± standard deviation (SD). The statistical software SPSS 13.0 (SPSS, Inc., Chicago, IL, USA) was used for statistical analysis. Paired comparisons were performed by the Student’s t-test. P<0.05 was considered to indicate a statistically significant difference.

## Results

### mRNA levels detected by qPCR

**Figure 2:**
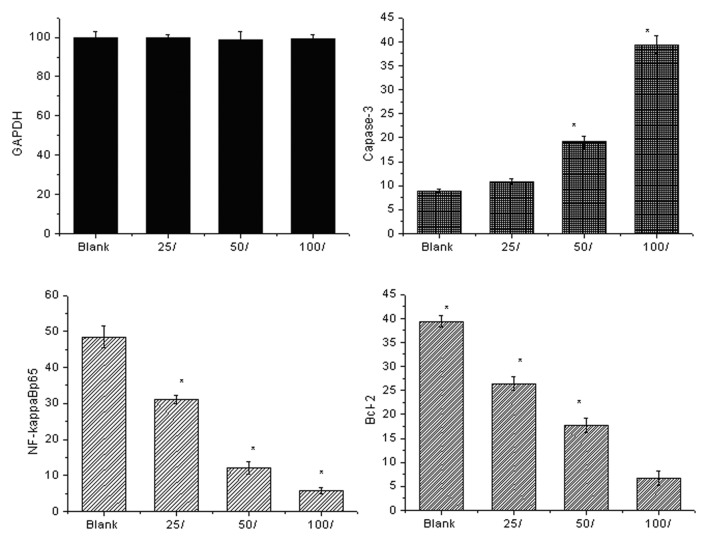
Relative mRNA levels (%) vs. glyceraldehyde 3-phosphate dehydrogenase (GAPDH). 7,8-Dihydroxycoumarin concentrations: 25, 50 and 100 *μ*mol/l. Blank: control cells. GAPDH: housekeeper gene. As 7,8-dihydroxycoumarin was used in a series of dilutions, i.e. 25, 50 and 100 *μ*mol/l, anti-apoptotic signaling was inhibited and pro-apoptotic signaling was activated. At 50.0 *μ*mol/l, the anti-apoptotic NF-κBp65 and Bcl-2 levels decreased 0.17 (8.9/52.6)-fold and 0.22 (9.5/42.3)-fold, respectively. Pro-apoptotic caspase-3 was upregulated 4.78 (38.7/8.1)-fold. ^*^P<0.05, compared with the control (n=3).

Fig. 2 shows the expression levels of the cell signaling molecules detected by qPCR. Prior to 7,8-dihydroxycoumarin treatment, the control cells expressed high levels of anti-apoptotic NF-κBp65 and Bcl-2 mRNAs and a low level of pro-apoptotic caspase-3. As 7,8-dihydroxycoumarin was used in a series of dilutions (25, 50 and 100 *μ*mol/l), the anti-apoptotic signaling was inhibited and the pro-apoptotic signaling was activated. The anti-apoptotic NF-κBp65 mRNA expression levels decreased 0.64 (31.04/48.5)-, 0.25 (12.1/48.5)- and 0.12 (5.82/48.5)-fold, respectively; and the levels of Bcl-2 mRNA decreased 0.67 (26.4/39.4)-, 0.45 (17.7/39.4)- and 0.17 (6.7/39.4)-fold, respectively. The pro-apoptotic caspase-3 increased 1.23 (10.9/8.9)-, 2.14 (19.1/8.9)-, and 4.43 (39.4/8.9)-fold, respectively. The pro-apoptotic induction effect of 7,8-dihydroxycoumarin is concentration-dependent.

### Protein levels detected by western blotting

**Figure 3:**
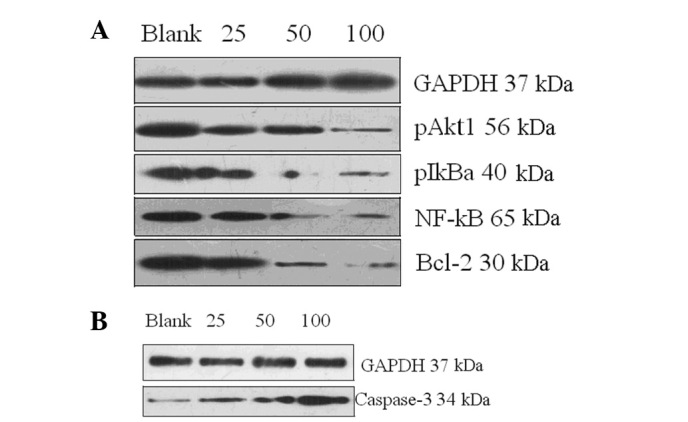
Blots of (A) anti-apoptotic and (B) pro-apoptotic proteins at 48 h. 7,8-Dihydroxycoumarin concentrations: 25, 50 and 100 *μ*mol/l; blank: control cells; glyceraldehyde 3-phosphate dehydrogenase (GAPDH): housekeeper gene; pAkt1, phospho-Akt1; pIκBα, phospho-IκBα.

Fig. 3 shows the expression of the cell signaling molecules detected by western blotting. Prior to treatment with 7,8-dihydroxycoumarin, the control cells expressed high levels of anti-apoptotic pAkt1, pIκBα, NF-κBp65 and Bcl-2 proteins and a low-level of pro-apoptotic caspase-3 protein. As 7,8-dihydroxycoumarin was used in a series of dilutions (25, 50 and 100 *μ*mol/l), the anti-apoptotic signaling was inhibited (Fig. 3A) and the pro-apoptotic signaling was upregulated (Fig. 3B).

Table I presents the complete gray scales of the blots shown in Fig. 3 to represent the total levels of the detected proteins. The anti-apoptotic pAkt1 protein blot grayscales were 36.5, 18.1 and 7.3 vs. 52.4 (each 7,8-dihydroxycoumarin dose vs. control), respectively; the pIκBα blot grayscales were 13.7, 7.6 and 4.3 vs. 42.2, respectively; the pNF-κBp65 blot grayscales were 23.3, 12.6 and 5.08 vs. 44.5; the Bcl-2 blot grayscales were 23.6, 17.9 and 5.92 vs. 38.5; and the pro-apoptotic caspase-3 blot grayscales were 7.61, 16.1 and 27.8 vs. 5.8, respectively. The pro-apoptotic induction effect of 7,8-dihydroxycoumarin is concentration-dependent.

### Cell proliferation

**Figure 4:**
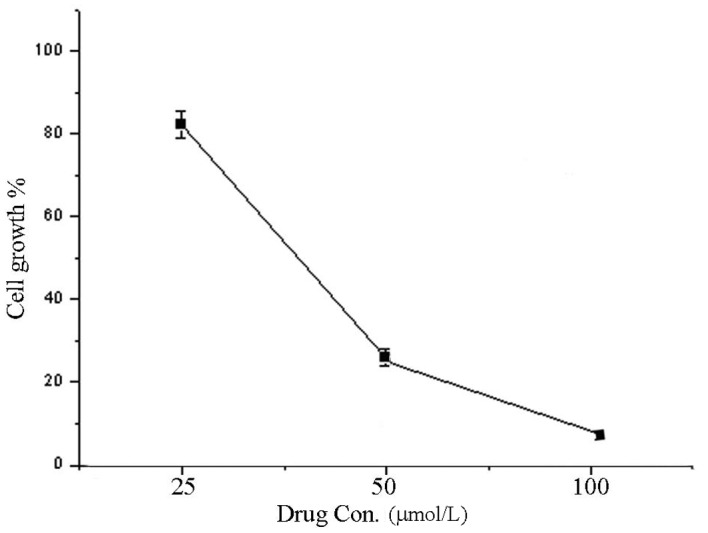
Cell proliferation of A549 lung adenocarcinoma cells treated with different concentrations of 7,8-dihydroxycoumarin (25, 50 and 100 *μ*mol/l) for 48 h (n=3).

Fig. 4 illustrates cell viability at 48 h. The proliferative activity of A549 cells treated with 7,8-dihydroxycoumarin decreased and was significantly lower compared with that of the control cells (83.7, 27.2 and 9.5 vs. 100%, respectively; P<0.05 for each). 7,8-Dihydroxycoumarin inhibited tumor cell proliferation in a concentration-dependent manner.

## Discussion

Inhibition of the Akt/NF-κB pathways results in the upregulation of pro-apoptotic Fas/APO-1, FasL, Bax (17), caspase-8, caspase-3 and cyt *c*, with simultaneous downregulation of NF-κBα, Akt, Bcl-2 and Bcl-xL (18). In the present study, we used 7,8-dihydroxycoumarin to treat A549 lung adenocarcinoma cells and then performed qPCR and western blotting to detect the ability of 7,8-dihydroxycoumarin to change the levels of anti-apoptotic pAkt, pIκBα, pNF-κB p65 and Bcl-2, as well as pro-apoptotic caspase-3.

Prior to treatment with 7,8-dihydroxycoumarin, there is an overexpression of Akt1 phosphorylated in control cells (19). Hyperactivated pAkt1 has serine-threonine protein kinase activity and triggers the cascaded enzymes, resulting in an increased phosphorylation of IκBα at serines 32 and 36. pIκBα was disassociated from the IκBα/NF-κB complex, resulting in a release of pNF-κB causing an increase of NF-κBp65 at the mRNA and protein levels. The hyperactivated pAkt1 also causes anti-apoptotic Bcl-2 to be maintained at high mRNA and protein levels, resulting in the sustained proliferation of the A549 control cells.

The use of 7,8-dihydroxycoumarin to treat A549 cells resulted in a marked downregulation of pAkt1 and pIκBα, as well as NF-κBp65 at the mRNA and protein levels. The downregulation of pAkt1 indicates that the serine-threonine protein kinase activity of Akt was reduced. Subsequently, the phosphorylation of IκBα was reduced. Thus the IκBα/NF-κB complex inhibited the release of NF-κB, resulting in a reduction in NF-κBp65 levels. As the serine-threonine protein kinase activity of Akt was reduced, anti-apoptotic Bcl-2 was simultaneously downregulated, so that the suppression of the apoptosis of A549 cells was reduced; therefore, apoptosis was facilitated.

The downregulation of pAkt1 and NF-κBp65 demonstrated that the signal amplification and transduction pathways were efficiently suppressed. Accordingly, the pro-apoptotic caspase-3 expression was increased. As reported in previous studies, upregulated caspase-3 inhibits IKK2 (20,21) in necrotized or apoptotic cancer cells, resulting in a further reduction in the phosphorylation of IκBα, causing the NF-κBp65 level to be further reduced. The upregulated caspase-3 also directly inhibits the NF-κBp65 protein (22), causing a secondary downregulation of NF-κBp65 in apoptotic cancer cells. Therefore, the NF-κBp65 signaling was markedly suppressed in A549 cells in the present study. The apoptotic A549 cells were observed to undergo reduced proliferation. The MTT assay results also demonstrated that the proliferation of A549 cells was significantly inhibited by 7,8-dihydroxycoumarin. In addition, the pro-apoptotic induction effect of 7,8-dihydroxycoumarin was concentration-dependent.

In conclusion, 7,8-dihydroxycoumarin inhibits the proliferation of A549 human lung adenocarcinoma cells and induces their apoptosis via Akt/NF-κB signaling suppression in a concentration-dependent manner. Akt and NF-κB may be targets for the treatment of lung adenocarcinoma. 7,8-Dihydroxycoumarin may be a candidate naturally occurring drug for the treatment and prevention of lung adenocarcinoma.

7,8-Dihydroxycoumarin, as an extract of naturally occurring plants, is safe and has a high efficacy. Therefore, it may be used in the clinic to treat lung carcinoma.

## Figures and Tables

**Table I:** Gray scales of the western blots (48 h, %/GAPDH).

Protein blot	Cell control	7,8-Dihydroxycoumarin concentration (*μ*mol/l)		
		25	50	100
GAPDH (37 kDa)	100.30	101.40	99.50	102.20
pAkt1 (56 kDa)	52.40	36.50	18.10	7.30
pIκBα (40 kDa)	42.20	13.70	7.60	4.30
NF-κBp65 (65 kDa)	44.50	23.30	12.60	5.08
Bcl-2 (30 kDa)	38.50	23.60	17.90	5.92
Caspase-3 (34 kDa)	5.80	7.61	16.10	27.80

GAPDH, glyceraldehyde 3-phosphate dehydrogenase; pAkt1, phospho-Akt1; pIκBα, phospho-IκBα.
